# Management of Cesarean Scar Pregnancy: A Single-Institution Retrospective Review

**DOI:** 10.1155/2018/6486407

**Published:** 2018-03-05

**Authors:** P. Giampaolino, N. De Rosa, I. Morra, A. Bertrando, A. Di Spiezio Sardo, B. Zizolfi, C. Ferrara, L. Della Corte, G. Bifulco

**Affiliations:** ^1^Department of Public Health, University of Naples Federico II, Via S. Pansini 5, 80131 Naples, Italy; ^2^Department of Neuroscience, Reproductive Sciences and Dentistry, University of Naples Federico II, Via S. Pansini 5, 80131 Naples, Italy

## Abstract

**Objective:**

Cesarean scar pregnancy (CSP) is a rare condition that occurs when the pregnancy implants in a cesarean scar. An early diagnosis and a proper management are fundamental to prevent maternal complications. We review and discuss the different treatment employed in our unit to reduce morbidity, preserve fertility, and predict possible complications.

**Methods:**

The reported treatment has been expectant management, operative hysteroscopy approach, and intramuscular injection of 50 mg methotrexate (MTX), followed by cervical dilation and manual vacuum aspiration (D&S) with a Karman cannula under ultrasound guidance, uterine artery embolization (UAE), and manual vacuum aspiration under ultrasound guidance and uterine artery embolization before surgical laparotomic resection.

**Results:**

Complications were more frequent in women with a history of three or more cesarean section deliveries and with a myometrial thickness thinner than 2 mm. MTX and D&S treatment appear to be most effective and safe at the early age of pregnancy, while UAE and D&S are related to the highest risk of complication in any age of pregnancy.

**Conclusion:**

An appropriate preoperative diagnostic evaluation, the identification of cases at higher risk, and those eligible for a conservative treatment are fundamental to reduce complications.

## 1. Introduction

Cesarean scar pregnancy (CSP) is a rare occurrence consisting in the implantation of the gestational sac in the hysterotomy scar [1, 2]. A recent review of the literature identified 751 cases of CSP [3]. The incidence of CSP has been estimated to range from 1/1008 to 1/2500 of all cesarean deliveries (CD) [4, 5] and in 72% of cases occurs in women who have had more than 2 CDs [6–10].

The exact pathogenic mechanisms are still unclear but CSP is believed to occur when a blastocyst implants on fibrous scar tissue within a wedge-shaped myometrial defect in the anterior lower uterine segment, by the site of a prior cesarean scar.

Due to the increase of CDs the incidence of CSP is rising. This condition can be dangerous for the women because of the related complications such as placenta previa or accreta, uterine rupture, and hemorrhage, leading to increased maternal morbidity and mortality. Therefore, an early diagnosis is crucial to improve the proper management. Several authors have reported sonographic criteria to aid in a diagnosis of CSP [3, 5, 11]. Despite the progress made in ultrasonography and radiological imaging methods, the optimal management remains to be determined.

Considering the lack of unique guidelines and management protocols, the aim of our study was to analyze the different methods of treatment for CSP offered by our team and identify the criteria on which to base the choice of the best approach for the women.

## 2. Material and Methods

This is a retrospective review of a case series of 45 pregnant women between 6 and 13 weeks, referred to our Department from 2013 to May 2017, with a diagnosis of CSP.

All clinical and anamnestic data were extracted from medical records of the patients.

The CSP was diagnosed by transvaginal ultrasound, according to diagnostic criteria as reported by several authors [3, 5, 11].

The following eligibility criteria had to be met: (1) the ultrasound images confirming the diagnosis were available and the gestational/chorionic sac was clearly visible as well as the entire uterus with its fundic and cervical contour; (2) adequate follow-up period was tight and registered.

All women gave written informed consent. All procedures performed in the study were in accordance with the ethical standards of the institutional and/or national research committee and with the 1964 Helsinki Declaration and its later amendments or comparable ethical standards. The study was not registered at the clinical trial registry and IRB approval was not obtained as it is a retrospective study that we did not change or experience but merely observed and collected data.

In 12 cases, the ultrasound showed a very thin or absent myometrial layer (<2 mm) between gestational sac and bladder; these women underwent MRI with contrast medium for suspected placental invasion of the bladder.

The treatment modalities in our experience are as follows:

- Expectant management (EM)
- Hysteroscopic resection of gestational tissue (HSC) performed under general anesthesia. The cervix was dilated up to 10 mm with Hegar's dilators, introducing a continuous flow 9 mm bipolar resectoscope with a 4 mm loop (Versapoint II, Gynecare; Ethicon, Somerville, NJ, USA). The setting of the electrosurgical generator was Versapulse modality with 110 W and 80 DES. Saline solution was used for distention and irrigation of the uterine cavity and the intrauterine pressure was automatically controlled by Endomat [12]
- Intramuscular injection of a single dose 50 mg of MTX, following 48 hours by cervical dilatation with 1 mg of vaginal Gemeprost dispensed 3 hours before the manual vacuum aspiration with a Karman cannula (D&S) under ultrasound guidance (MTXii + D&S)
- Uterine artery embolization (UAE) and manual vacuum aspiration (D&S) under ultrasound guidance following the procedure described above (UAE + D&S)
- UAE and surgical laparotomic resection (UAE + Surg).

Complications, like uterine rupture or profuse bleeding, were treated by means of a Foley catheter inflated with 30–40 cc in the isthmus region for 24 hours. The severe bleeding was considered when we observed a drop in hemoglobin (Hb) levels greater than 5 units and/or a reduction of hematocrit (Hct) percentage greater than 10%. In the presence of Hb levels below 6 mg/dl and/or Hct under 20%, if necessary, blood cell transfusion was administered.

All women treated with conservative management underwent clinical and instrumental follow-up one week and one month later.

All statistical analyses were performed with SPSS version 20.0 (SPSS Inc., Chicago, IL, USA). Comparisons between unpaired groups were made by Student's *t*-test or one-way analysis of variance (ANOVA) for continuous variables and *χ*^2^ test for categorical variables.

Receiver operating characteristic (ROC) curve analysis and logistic regression analysis were used to evaluate factors predicting treatment complication. *p* < 0.05 was considered statistically significant.

## 3. Results

Women' ages ranged from 26 to 42 years (mean: 33.68 ± 3.52 years). Clinical women's data in relation to treatment modalities are shown in Table 1.

All 45 women had an empty uterine cavity at ultrasound scan, and the gestational sac was detected in the hysterotomy scar, adjacent to the bladder. HCG levels were recorded.

All women had a history of prior uterine surgery. As for the number of cesarean sections before the CSP, of the 45 women, 14 (31.1%) had had one, 17 (37.8%) had had two, 10 (22.2%) had had three, and 4 (8.9%) had had four CDs.

Thirty-four patients (75.6%) showed a low gestational age (≤8 weeks) at the time of diagnosis and treatment.

According to ultrasound findings, which focused on the myometrial thickness and the presence of a vascular pattern of the cesarean section scar, we stratified patients into three risk classes (the first class results of high risk and class 3 results low risk):

- Class 1: myometrial thickness ≤ 2 mm and high vascular pattern
- Class 2: myometrial thickness > 2 mm and high vascular pattern
- Class 3: myometrial thickness > 2 mm and normal vascular pattern.

The color Doppler showed a rich vascular pattern in the cesarean section scar in 36 cases (80.00%) and a myometrial thickness ≤ 2 mm in 12 patients (26.7%), so that 12 patients (26.7%) were assigned to the first class risk group, 24 patients (53.33%) to risk class 2, and the last 9 patients (20.0%) to risk class 3.

**Figure 1:**
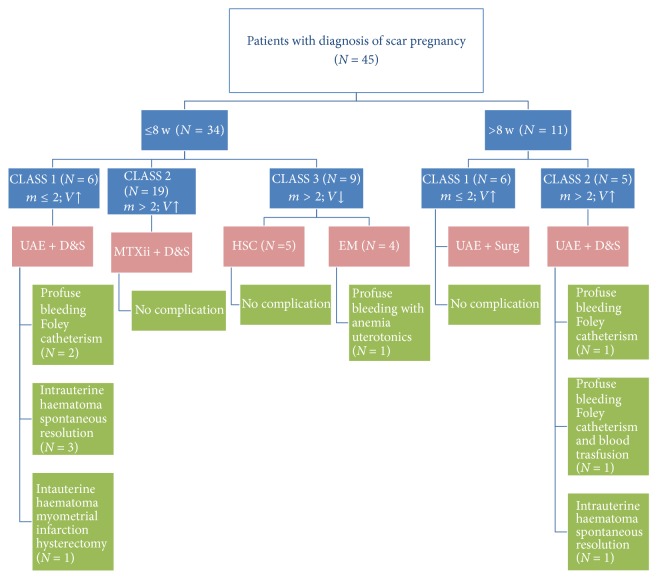
*Flow chart of patients: class risk, treatment modality, and postoperative complications*. According to ultrasound findings that focused on myometrial thickness and presence of a vascular pattern of the cesarean section scar, we stratified patients into tree risk classes: class 1: myometrial thickness ≤ 2 and high vascular pattern; class 2: myometrial thickness > 2 and high vascular pattern; class 3: myometrial thickness > 2 and normal vascular pattern. W: weeks of gestation at diagnosis; UAE: uterine artery embolization; D&S: dilatation and suction; MTX ii: methotrexate intramuscular injection; HSC: hysteroscopic resection; EM: Expectant Management; Surg: surgery (laparotomic edge excision or hysterectomy).

According to risk classes and gestational age, the modality treatment was summarized in Figure 1.

In 4 cases (8.9%), the EM approach was adopted, in consideration of the low gestational age (6 weeks) with mean myometrial thickness > 3 mm and the absent rich vascular pattern (Table 1); during the recovery, the patient had a complete miscarriage. In these cases, the ultrasound scan after metrorrhagia failed to detect intrauterine material, even in the scar area. According to our retrospective analysis, these patients have been grouped in risk class 3. In one case (25%) the profuse bleeding due to complete miscarriage caused a drop in hemoglobin levels to 7.2 g/dl and the patient was treated with uterotonics. After 3 days, all the patients were discharged in good physical conditions; the follow-up with ultrasound scan and biochemical testing after 1 week and 1 month did not detect any complications.

Five patients (11.1%) in risk class 3 and with gestational age < 8 weeks were treated with HSC. None of the complications were observed at follow-up in these patients.

Nineteen women (42.2%) within <8 weeks gestational age, myometrial thickness > 2 mm, and rich vascular pattern were treated with intramuscular injection of 50 mg MTX and, after 48 hours, D&S under ultrasound guidance. No postoperative complications were observed at the 1-week and 1-month follow-up (Table 1). According to our retrospective analysis, these patients have been grouped in risk class 2.

Eleven women (24.4%) were treated with UAE and D&S under ultrasound guidance; 6 of them had gestational age <8 weeks, myometrial thickness ≤ 2 mm, and a rich vascular pattern (risk class 1). Two patients presented with profuse vaginal bleeding and mechanical hemostasis with a Foley catheter for 24 hours was necessary. Three patients presented intrauterine haematoma 24 h after treatment with spontaneous resolution after about 40-day follow-up. In only one case, severe intraoperative bleeding caused haematoma and hemorrhagic myometrial infarction in the scar area, which was treated by urgent subtotal hysterectomy, prophylactic salpingectomy, and ovarian conservation (Table 2).

The other 5 patients performing UAE and D&S had gestational age ≥ 8 weeks with a rich vascular pattern and myometrial thickness > 2 mm (risk class 2). Two patients presented profuse vaginal bleeding and mechanical hemostasis with a Foley catheter for 24 hours was necessary, and one of them was treated with 2 units of red blood cells because hemoglobin levels were below 6 mg/dl. One patient presented intrauterine haematoma 24 h after treatment with spontaneous resolution after about 40-day follow-up.

All women with more advanced gestational age showed an increased vascularization pattern at color Doppler evaluation independently from myometrial thickness, so that we have no patients with advanced gestational age belonging to class risk 3.

Six women (13.3%) with gestational age > 8 weeks belonging to class risk 1 were treated with UAE and surgery. Four women underwent edge excision of CSP by laparotomic hysterotomy with successful repair of the myometrial scar. Two patients underwent hysterectomy with prophylactic salpingectomy and ovarian conservation for the absence of myometrial thickness at laparotomy. No further complications were observed in follow-up.

A significant difference between treatment's groups have been shown in gestational age, number of previous CDs, myometrial thickness, initial serum *β*-hCG level, or vascular pattern of ultrasonographic findings (Table 1). Only mean women's age did not differ between groups.

The occurrence of complication significantly differed from group (*p* ≤ 0.001); UAE + D&S showed the highest percentage of complications.

In our study, complications after treatment of CSP were observed in 22.2% of cases (10 of 45 women). Five patients (11.1%) had profuse bleeding and another 5 women (11.1%) had haematoma, but in only one case we observed myometrial infarction. Only this woman was treated with radical surgery. In other cases, conservative management was effective.

Table 2 lists the results of univariate analysis of risk factors for complication during treatment. It is clear that in the complication group a significantly higher number of previous CDs, a smaller myometrial thickness, a higher percentage of UAE + D&S, and the higher proportion of cases belong to class risk 1 or 2 (*p* < 0.005).

ROC curve analysis and logistic regression were used to evaluate risk factors capable of predicting treatment complication, including number of previous cesareans, myometrial thickness, treatment modality, and class risk.

**Figure 2:**
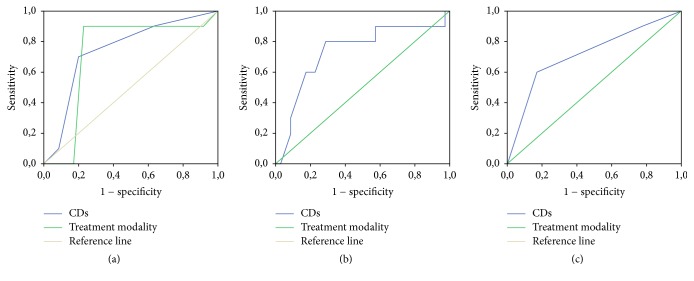
Receiver operating characteristic (ROC) curve analysis of risk factors for treatment complications. (a) The AUCs for number of cesarean deliveries (CDs) and treatment modalities; (b) myometrial thickness; (c) risk class.

The areas under curve (AUC), for the number of previous cesarean deliveries, treatment modality myometrial thickness, and class risk, were 0.75 (*p* = 0.019; CI 95%: 0.57–0.92) (Figure 2(a)); 0.72 (*p* = 0.03; CI (95%): 0.54–0.91) (Figure 2(a)); 0.74 (*p* = 0.02; CI (95%): 0.54–0.93) (Figure 2(b)); and 0.72 (*p* = 0.04 CI (95%): 0.52–0.91) (Figure 2(c)), respectively.

**Figure 3:**
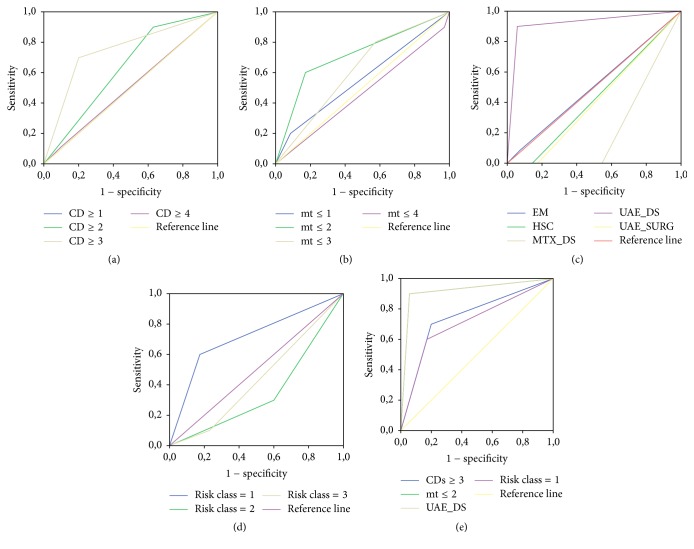
Receiver operating characteristic (ROC) curve analysis of risk factors for primary treatment complications. (a) For number of cesarean deliveries, a cutoff of 3 was the preferable indicator. (b) A 2 mm cutoff was the preferable myometrial thickness indicator. (c) The UAE + D&S was the major indicator of treatment complication occurrence. (d) Considering risk class the cutoff value was risk class = 1. (e) When the four cutoff variables were also compared the UAE & DS was the risk factor with the higher AUC.

For the number of cesarean deliveries, a cutoff of 3 was the preferable indicator. The AUCs were, for CDs ≥ 1, 0.50 (*p* = 1.0; CI (95%): 0.30–0.71); CDs ≥ 2, 0.64 (*p* = 0.19; CI (95%): 0.46–0.82); CDs ≥ 3, 0.75 (*p* = 0.02; CI (95%): 0.57–0.93); and CDs ≥ 4, 0.51 (*p* = 0.95; CI (95%): 0.30–0.71) (Figure 3(a)).

A 2 mm cut off was the preferable myometrial thickness indicator The AUCs were for a myometrial thickness ≤ 1, 2, 3, and 4 mm, respectively, 0.56 (*p* = 0.59; CI (95%): 0.34–0.77); 0.71 (*p* = 0.04; CI (95%): 0.52–0.91); 0.61 (*p* = 0.28; CI (95%): 0.43–0.80); and 0.46 (*p* = 0.73; CI (95%): 0.25–0.68) (Figure 3(b)).

The UAE + D&S was the major indicator of treatment complication occurrence. The AUCs for each treatment were for EM 0.51 (*p* = 0.95; CI (95%): 0.30–0.71); for HSC 0.43 (*p* = 0.50; CI (95%): 0.24–0.62); for MTX + D&S 0.23 (*p* = 0.01; CI (95%): 0.09–0.36); for UAE + D&S 0.92 (*p* ≤ 0.001; CI (95%): 0.80–1.0); and for UAE + SURG 0.41 (*p* = 0.41; CI (95%): 0.23–0.6) (Figure 3(c)).

Considering risk class, the cut off value was risk class = 1. The AUCs for each class were for risk class 1: 0.71 (*p* = 0.04; CI (95%): 0.52–0.91); for risk class 2: 0.35 (*p* = 0.15; CI (95%): 0.16–0.54); for risk class 3: 0.44 (*p* = 0.54; CI (95%): 0.24–0.63) (Figure 3(d)).

The four cuts off variables were also compared by ROC analysis (Figure 3(e)).

In the binary logistic regression model, only performing UAE + D&S was a significant risk factor for complication, with odds ratio of 119.6 (CI [95%]: 18.03–793.16, *p* ≤ 0.001) (Table 3).

## 4. Discussion

In the present study, 77,8% (*N* = 35) of patients with CSP were successfully treated without any complications. Among patients who reported complications, only 1 had severe complications requiring destructive surgery, whereas in 90.0% of the cases the complication was manageable by mechanical hemostats and/or blood transfusion. Our complication rate is lower than the one reported in the literature. The selection method of patients, adopted in our institution, allows reducing treatment failure.

In their review, Timor-Tritsch and Monteagudo [3] analyzed the different therapeutic approaches of the CSP. The authors found about 31 primary treatment methods for the 751 CSP cases analyzed: some of them predict an essentially surgical approach with hysteroscopy, cervical dilation and curettage (D&C), excision of the CSP, hysterectomy or embolization of the uterine arteries, and other exclusively medical approaches with methotrexate (MTX); the majority of cases combined a surgical and a pharmacological approach [3]. The authors found that the rate of complications requiring a second intervention was very high (45%) [3]. The four treatments with the highest number of complications were those involving the use of MTX alone (62%), D&C (62%), UAE (47%), and the administration of intramuscular MTX combined with D&C (86%).

In our experience, most of the women were treated with a combined approach MTX and D&S or UAE and D&S. In contrast to previously reported data [3], the combined approach of MTX + D&S results were effective and safe, with no complications, in 100% of cases. This is probably due to the patient selection mode; in particular, our protocol consisted in limiting this treatment to women with earlier gestational periods eligible for the use of MTX [13].

On the other hand, in our study group, the combined approach UAE + D&S results in having a higher number of complications (90%) both in lower than in high gestational age. So, undergoing UAE + DS results in a specific significant risk factor in terms of complications.

Our data confirm that the number of previous cesareans, the myometrial thickness, and the class risk are specific factors in complication's occurrence. On the other hand, our data lack in demonstrating that the gestational age and the presence of a rich vascular pattern are specific risk factors. This lack of evidence may be attributable to the fact that the evaluation of the vascular pattern, in our study, was subjective and not performed by VOCAL index so that may be overestimated. Moreover, this is a retrospective study and both gestational age and vascular pattern have been considered as selective criteria in the treatment modality limiting their effects on statistical analysis.

However, color Doppler evidence of a high-speed peritrophoblastic flow and low resistance near the hysterotomy scar [11] has been mentioned as important factors in the differential diagnosis with the abortion in expulsion and also as a prognostic index for possible treatment complications. Particularly, Chou et al. in 2004 [14] and Timor-Tritsch et al. in 2012 [15] described the use of a 3D system to quantify peritrophoblastic neovascularization and study its own characteristics to identify possible complications and/or evaluate the outcome of conservative CSP therapy. The sonographic criteria contribute to a correct diagnosis in 86.4% of cases.

To date, hysteroscopy represents a widely accepted method for the management of several gynecological conditions [16–20] or even to confirm ultrasound findings [21–23].

In this regard, several authors in recent years have described the successful hysteroscopic treatment of CSPs in combination with another surgical approach [24] or medical procedure [12, 25] or alone [26] in early gestational age.

In our study group, we have no complication after hysteroscopic approach, so this treatment appears safe and effective. Taking into account that we have reported only 5 cases considered of low-risk class, another study should confirm our data.

Some studies report [27, 28] the occurrence of complications before diagnosis; for this reason the expectant management is not considered an approach of choice, because the histopathologic features of CSP prevent a complete detachment of the gestational sac from the home plant, exposing the pregnant woman to a high-risk hemorrhagic event [29, 30]. In our study group, only 4 women underwent an expectant management with a complication rate of 25%.

However, in a recent review, Birch Petersen et al. recommended five approaches for treating CSP depending on availability, the severity of symptoms, and surgical skills. In this paper, the authors support an interventional rather than a medical approach. They have reported the use of laparoscopic surgical treatment as effective in selected cases and when performed by laparoscopist surgeons with specific expertise in the subject [31]. In our study, laparoscopic treatment was not performed because the patients undergone surgery were all at high risk (class 1) and with multiple previous cesarean sections. In our experience, and according to literature, the presence of previous laparotomies has led us to prefer the laparotomic approach to laparoscopic one [32, 33].

Among the most important diagnostic criteria to evaluate the risk of complications, there are myometrial thickness and the numbers of previous CDs [3, 34]. In our series, a smaller myometrial thickness and a high number of previous CDs were related to the high number of complications.

The combination of MTX + D&C appears to be the most effective and safe treatment for women in the early stages of pregnancy, whereas UAE and D&S result in a significant specific risk factor for complication independent of gestational age.

In conclusion, our study shows an overall complication rate of 22.2%, lower than that reported in the literature (44.1%) [3]. The complication rate is overall reduced by an appropriate preoperative diagnostic ultrasound evaluation of the individual case, which points not only to the correct diagnosis of CSP, but also to the identification of cases at higher risk of complications and those eligible for a conservative treatment. Our data indicate that a treatment combining MTX and D&S or HSC appears to be effective and safe in pregnancies with early gestational age. This underlines the importance of a timely diagnosis to minimize side effects and complications.

The standard treatment has not been established in the management of scar pregnancy yet. However, the correct diagnosis and the personalized evaluation of risk factors could support physicians in making the best choice in terms of safety and efficacy.

## Figures and Tables

**Table 1:** Clinical patient's data in relation to treatment modality.

	EM (*N* = 4)	HSC (*N* = 5)	MTX ii and D&S (*N* = 19)	UAE and D&S (*N* = 11)	UAE and Surg. (*N* = 6)	*p*-value
Gestational age (wks)^*∗*^	6 ± 0,82	6,8 ± 0,84	7,21 ± 0,63	8,0 ± 1,73	10,17 ± 1,17	≤0.001
Women age (y)^*∗*^	34,5 ± 3,11	32,6 ± 4,93	32,68 ± 3,92	34,55 ± 2,62	35,67 ± 1,75	0.31
previous CDs (no.)^*∗*^	1,25 ± 0,50	1,4 ± 0,55	1,68 ± 0,58	2,64 ± 0,71	3,5 ± 0,55	≤0.001
Myometrial thickness^*∗*^ (mm)	3,78 ± 0,33	3,36 ± 0,35	3,07 ± 0,63	1,96 ± 0,99	1,08 ± 0.50	≤0.001
Rich vascular pattern^§^	0	0	19 (100)	11 (100)	6 (100)	≤0.001
BhCG (mIU/mL)^*∗*^	5890,5 ± 1284,15	9756 ± 3286,50	11790 ± 4904,67	49976,54 ± 686636,34	16909,83 ± 48712,1	≤0.001
Complication^§^	1 (25)	0	0	9 (81,8)	0	≤0.001

EM: expectant management; HSC: hysteroscopic resection; MTH ii: methotrexate intramuscular injection; D&S: dilatation and suction; UAE: uterine artery embolization; Surg: surgery. ^*∗*^Data are shown as mean ± SD; statistical analysis performed by one-way analysis of variance. ^§^Data are shown as no. (percentage); statistical analysis performed by *χ*^2^ test.

**Table 2:** Complications after treatment of cesarean scar pregnancy.

	Complications (*N* = 10)	No complications (*N* = 35)	*p* value
Gestational age (wks)^*∗*^	7.5 ± 1.43	7.69 ± 1.60	0.56
Women age (y)^*∗*^	34.7 ± 2.71	33.4 ± 3.70	0.31
previous CDs (no.)^*∗*^	2.70 ± 0.82	1.91 ± 0.92	0.019
Myometrial thickness^*∗*^ (mm)	1.94 ± 1.12	2.8 ± 0.97	0.018
Rich vascular pattern^§^	9 (90)	27 (77)	0.37
BhCG (mIU/mL)^*∗*^	35034 ± 52750	42434 ± 67085	0.75
Treatment^§^			≤0.001
EM	1 (10)	3 (8.6)	
HSC	0	5 (14.3)	
MTX + D&S	0	19 (54.3)	
UAE + D&S	9 (90)	2 (5.7)	
UAE + SURG	0	6 (17.1)	
Risk Class^§^			0.03
1	6 (60.0)	6 (17.1)	
2	3 (30.0)	21 (60.0)	
3	1 (10.0)	8 (22.9)	

EM: expectant management; HSC: hysteroscopic resection; MTH ii: methotrexate intramuscular injection; D&S: dilatation and suction; UAE: uterine artery embolization; Surg: surgery. ^*∗*^Data are shown as mean ± SD; statistical analysis performed by Student's *t*-test for unpaired data; ^§^Data are shown as no. (percentage); statistical analysis performed by *χ*^2^ test.

**Table 3:** Logistic regression models for factors predicting treatment complications.

Factor	Number of women	Univariate	*p* value
		OR (95% CI)	
Previous CDs ≥ 3	28	**3.80 (0.39–37.56)**	0.25
Previous CDs < 3	62		

Myometrial thickness ≤ 2	24	**2.078 (0.21–20,84)**	0.53
Myometrial thickness > 2	66		

Risk class = 1	24	**2,078 (0.21–20,84)**	0.53
Risk class = 2 and 3	66		

UAE + D&S	22	**119.60 (18.03–793.16)**	≤0.001
Other treatment	68		

CD: cesarean deliveries; UAE + D&S: uterine artery embolization and dilatation and suction; OR: odds ratio; CI: confidence interval.

## Additional Points

*Synopsis*. Methotrexate and D&C represented an effective treatment in case of early cesarean scar pregnancy (CSP); therefore an early diagnosis and identification of women at high risk reduce the complications.
